# Macrophage activation syndrome in patients with systemic juvenile idiopathic arthritis treated with tocilizumab

**DOI:** 10.1186/1546-0096-12-S1-P55

**Published:** 2014-09-17

**Authors:** Fabrizio De Benedetti, Rayfel Schneider, Sheila Weitzman, Clare Devlin, Kaori Daimaru, Shumpei Yokota, Syuji Takei, Angelo Ravelli

**Affiliations:** 1 IRCCS Ospedale Pediatrico Bambino Gesú, Rome, Italy; 2 The Hospital for Sick Children, Toronto, Canada; 3 Roche Products Ltd., Welwyn Garden City, UK; 4 Chugai Pharmaceutical, Tokyo; 5 Yokohama City University School of Medicine, Yokohama, Japan; 6 School of Health Science, Faculty of Medicine, Kagoshima University, Kagoshima, Japan; 7 University of Genoa and Istituto Giannina Gaslini, Genoa, Italy

## Introduction

Macrophage activation syndrome (MAS) is a severe, potentially fatal complication of systemic juvenile idiopathic arthritis (sJIA). Changes in therapies, including biologics, have been associated with the onset of MAS. Interleukin-6 (IL-6) plays a major pathogenic role in sJIA; data in animals suggest that high IL-6 levels contribute to the triggering of MAS [1]. Treatment with the IL-6 receptor inhibitor tocilizumab (TCZ) is highly effective in patients with sJIA [2].

## Objectives

To investigate the rates and features of MAS occurring during TCZ treatment in patients with sJIA.

## Methods

Data were collected from patients with sJIA treated with TCZ in the international phase 3 trial (TENDER), 4 clinical trials in Japan, and the Japanese postmarketing surveillance (JPMS) program. Reported MAS events or disease flares associated with alanine aminotransferase/aspartate aminotransferase (ALT/AST) elevations were collected. Worksheets with event information were assessed by an independent panel (2 pediatric rheumatologists, 1 pediatric hematologist with MAS expertise) overseen by the TENDER lead investigator. Cases were adjudicated as definite MAS, potential MAS, not MAS, or insufficient data.

## Results

The data set included 112 patients from TENDER (403.0 patient-years’ [PY] exposure to TCZ), 149 patients from the Japanese trials (326 PY), and 366 patients from the JPMS program (523.9 PY). Of 31 cases reviewed, 22 events were adjudicated as definite or potential MAS: 5 from TENDER (3 definite, 2 potential), 6 from the Japanese trials (3 definite, 3 potential), and 11 from the JPMS program (5 definite, 6 potential). The rates/100 PY of definite/potential MAS were 1.24 (95% CI, 0.4-2.90) in TENDER, 1.84 (95% CI, 0.68-4.00) in the Japanese trials, and 2.10 (95% CI, 1.05-3.76) in the JPMS program. Laboratory and clinical features most commonly contributing to the adjudication of the 11 definite MAS events were elevated ALT/AST in 11 (100%), thrombocytopenia in 10 (91%), elevated ferritin in 8 (73%), leukopenia in 7 (64%), neutropenia in 6 (55%), and fever in 6 (55%). All 11 events adjudicated as definite MAS met the preliminary MAS diagnostic guidelines [3]. All definite and potential MAS resolved, with the exception of MAS in a patient from the Japanese phase 3 study who died after respiratory/cardiac arrest.

## Conclusion

The use of TCZ does not appear to be associated with increased risk for MAS in sJIA. No unusual clinical or laboratory features were observed in these MAS cases.

## Disclosure of interest

F. De Benedetti Grant / Research Support from: Abbott, Pfizer, BMS, Roche, Novimmune, Novartis, SOBI, R. Schneider Consultant for: Roche, Novartis, S. Weitzman: None declared., C. Devlin Employee of: Roche Products Ltd., K. Daimaru Employee of: Chugai Pharmaceutical, S. Yokota Consultant for: Chugai (includes royalties), S. Takei Grant / Research Support from: BMS, Chugai, Eisai, Takeda, Speaker Bureau of: Chugai, taisyo-Toyama, Japan Blood Products, Santen, Takeda, Eisai, A. Ravelli Grant / Research Support from: Pfizer, Consultant for: Roche, Speaker Bureau of: Pfizer, AbbVie, BMS, Novartis.
